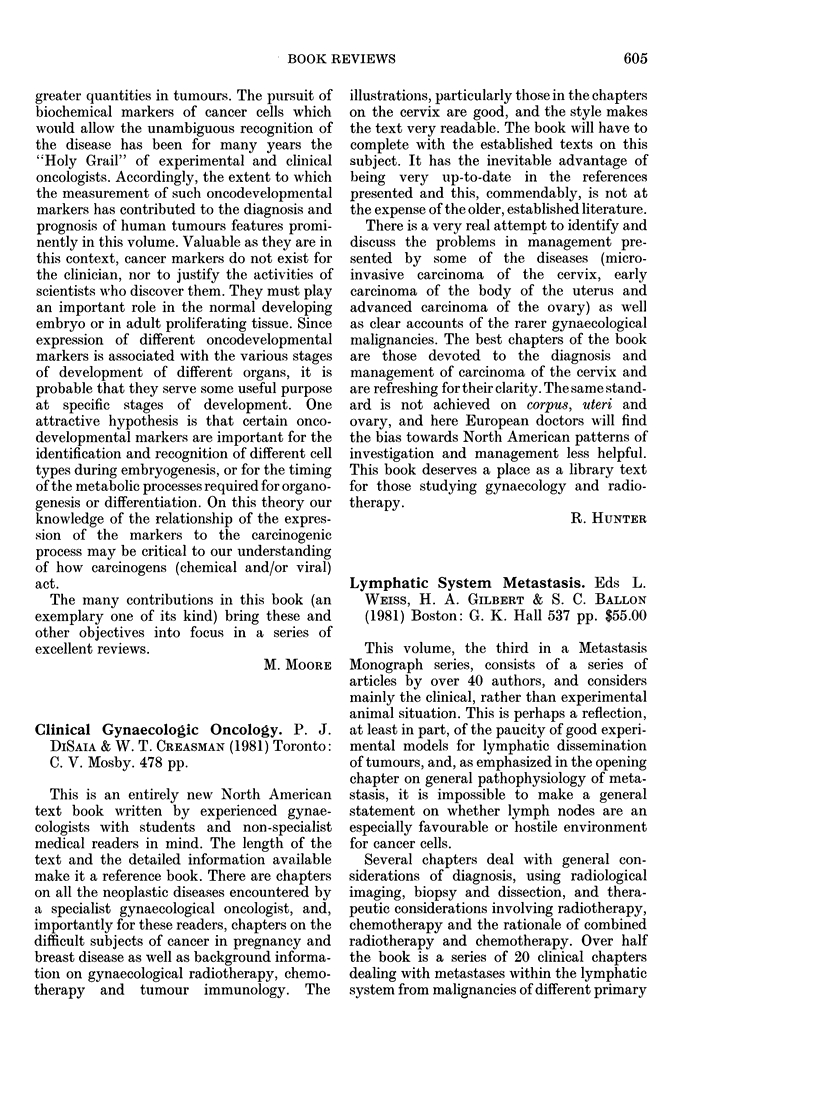# Clinical Gynaecologic Oncology

**Published:** 1981-10

**Authors:** R. Hunter


**Clinical Gynaecologic Oncology.** P. J. DISAIA & W. T. CREASMAN (1981) Toronto: C. V. Mosby. 478 pp.

This is an entirely new North American text book written by experienced gynaecologists with students and non-specialist medical readers in mind. The length of the text and the detailed information available make it a reference book. There are chapters on all the neoplastic diseases encountered by a specialist gynaecological oncologist, and, importantly for these readers, chapters on the difficult subjects of cancer in pregnancy and breast disease as well as background information on gynaecological radiotherapy, chemotherapy and tumour immunology. The illustrations, particularly those in the chapters on the cervix are good, and the style makes the text very readable. The book will have to complete with the established texts on this subject. It has the inevitable advantage of being very up-to-date in the references presented and this, commendably, is not at the expense of the older, established literature.

There is a very real attempt to identify and discuss the problems in management presented by some of the diseases (micro-invasive carcinoma of the cervix, early carcinoma of the body of the uterus and advanced carcinoma of the ovary) as well as clear accounts of the rarer gynaecological malignancies. The best chapters of the book are those devoted to the diagnosis and management of carcinoma of the cervix and are refreshing for their clarity. The same standard is not achieved on *corpus*, *uteri* and ovary, and here European doctors will find the bias towards North American patterns of investigation and management less helpful. This book deserves a place as a library text for those studying gynaecology and radiotherapy.

R. HUNTER